# Fenton-like Degradation of Methylene Blue on Attapulgite Clay Composite by Loading of Iron–Oxide: Eco-Friendly Preparation and Its Catalytic Activity

**DOI:** 10.3390/ma17112615

**Published:** 2024-05-29

**Authors:** Naveed Karim, Tin Kyawoo, Chao Jiang, Saeed Ahmed, Weiliang Tian, Huiyu Li, Yongjun Feng

**Affiliations:** 1 State Key Laboratory of Chemical Resource Engineering, Beijing Engineering Center for Hierarchical Catalysts, Beijing University of Chemical Technology, No. 15 Beisanhuan East Road, Chaoyang District, Beijing 100029, China; karimnaveed999@gmail.com (N.K.); tinkyawoo.mu@gmail.com (T.K.); 15222443969@163.com (C.J.); 2 Department of Chemistry, University of Chakwal, Chakwal 48800, Pakistan; saeed.ahmed@uoc.edu.pk; 3 College of Chemistry and Chemical Engineering, Tarim University, Alar 843300, China; 120100037@taru.edu.cn

**Keywords:** methylene blue, Fenton oxidation, adsorption kinetics, attapulgite, iron–oxide

## Abstract

The continuous discharge of organic dyes into freshwater resources poses a long-term hazard to aquatic life. The advanced oxidation Fenton process is a combo of adsorption and degradation of pollutants to detoxify toxic effluents, such as anti-bacterial drugs, antibiotics, and organic dyes. In this work, an activated attapulgite clay-loaded iron-oxide (A-ATP@Fe_3_O_4_) was produced using a two-step reaction, in which attapulgite serves as an enrichment matrix and Fe_3_O_4_ functions as the active degrading component. The maximum adsorption capacity (q_t_) was determined by assessing the effect of temperature, pH H_2_O_2_, and adsorbent. The results showed that the A-ATP@Fe_3_O_4_ achieves the highest removal rate of 99.6% under optimum conditions: 40 °C, pH = 3, H_2_O_2_ 25 mM, and 0.1 g dosage of the composite. The dye removal procedure achieved adsorption and degradation equilibrium in 120 and 30 min, respectively, by following the same processes as the advanced oxidation approach. Catalytic activity, kinetics, and specified surface characteristics suggest that A-ATP@Fe_3_O_4_ is one of the most promising candidates for advanced oxidation-enrooted removal of organic dyes.

## 1. Introduction

The sustainable management and distribution of water resources for a community’s health, sustainable agricultural growth, living things, food safety, and a clean drinking water supply management is crucial [1,2,3,4,5]. The sustainable management of water resources is seriously threatened by widespread urbanization, population growth, large-scale industrial outlet expansion, and inadequate water management practices [6,7,8]. A further major barrier to achieving these sustainable development goals is the removal of agricultural toxicants from textile outlets, such as weed killers, sprays, herbicides, germicides, and organic dyes [9,10,11]. Approximately 6500 tons of organic dyes are utilized globally each year, and these organic molecules are consistently present in water reservoirs for an extended period of time [12]. These chemicals are harmful to aquatic life and water plants, and they also increase the chance of cancer-related illnesses in people [13]. Amongst these dyes, methylene blue (MB) is a cationic azo-dye characterized by the molecular formula C_16_H_18_Cl_1_N_3_S·xH_2_O and a molecular weight of 319.85 g/mol in its anhydrous state [14]. MB is widely used in the textile and pharmaceutical industries as a coloring and staining agent. Its presence in water beyond the permissible limits can cause skin diseases, throat diseases, high blood pressure, gastrointestinal disorders, nausea, vomiting, and diarrhea [15].

To prevent aquatic disruptions, this harmful effluent must be removed from wastewater resources [16]. According to recent research, the Fenton process has the diverse ability to release free radicals into degraded dyes, making it more effective in degrading organic dyes than a straightforward adsorptive method [17]. The Fenton reaction primarily follows advanced oxidation processes (AOPs) induced by Fe ions, which degrade organic contaminants into simpler molecules efficiently. However, the classic Fenton oxidation method always comes with the formation of iron sludge, a low utilization of H_2_O_2_, and difficulty in catalyst recovery [18]. To circumvent these constraints, heterogeneous Fenton-like catalysts containing iron species have been developed as a potential technique due to their low energy consumption and great efficiency in removing pollutants [19]. Although heterogeneous Fenton catalysts are effective at degrading organic molecules, there are still challenges with catalyst separation and reuse, raising treatment costs. Furthermore, they are difficult and expensive to manufacture, complicating industrial manufacturing. As a result, there is an urgent need to produce low-cost, environmentally friendly Fenton catalysts with excellent and consistent catalytic activity [20,21].

Attapulgite (ATP) is a crystalline hydrated magnesium silicate mineral with a unique layer-chain crystal structure. It has many microporous channels and a relatively large surface area [22,23,24,25]. The distinctive composition of ATP gives it exceptional adsorption properties due to its hierarchical arrangement of pore sizes [26]. In addition, ATP can combine with metallic oxides to improve the degradation properties of several kinds of organic dyes, such as methyl-orange, bis-phenol, Rhodamine-D, Rhodamine-B, and 2,4-dichloro-phenol [27,28,29]. However, these functional catalysts are basically metallic oxides, which have not realized the effective use of attapulgite [30,31].

In this work, we have purified the raw ATP (R-ATP) using the co-precipitation method (P-ATP), and activated it using inorganic acids to fabricate activated ATP (A-ATP) with a higher surface area and compatibility. In addition, we have fabricated the iron–oxide/attapulgite (ATP@Fe_3_O_4_) composites of P-ATP, and A-ATP for the degradation of MB. Our investigation not only explores the efficacy of this hybrid material but also unravels the underlying mechanisms governing the heterogeneous Fenton reaction, and its applications in wastewater treatment.

## 2. Materials and Methods

### 2.1. Chemicals and Reagents

Fe_3_O_4_ nanoparticles_,_ HCl, H_2_O_2_, and MB were purchased from Shanghai Aladdin Bio-Chem Technology Co., Ltd. (Shanghai, China). All the reagents were of analytical grade and utilized without further processing. R-ATP was obtained from Gansu Western Attapulgite Research and Application Institute (Baiyin, China). All the solutions were prepared using deionized water.

### 2.2. Fabrication of Attapulgite Iron–Oxide Composite

**Figure 1:**
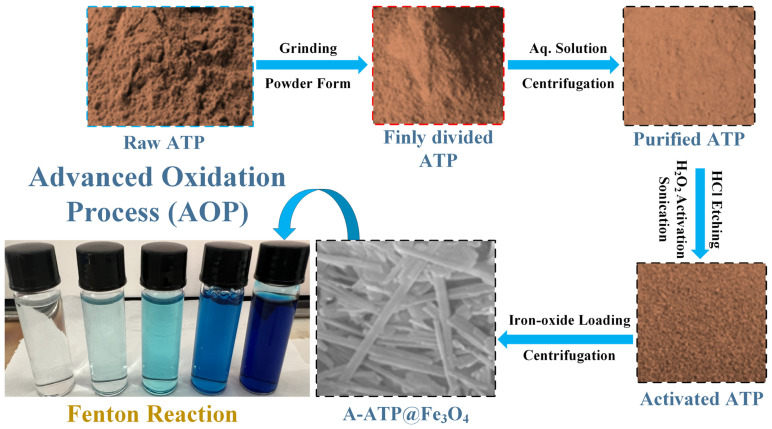
Fabrication Scheme of A-ATP@Fe_3_O_4_ Composite.

Initially, 10 g of R-ATP was thoroughly mixed with 300 mL of deionized water and stirred (magnetic stirring) for 2 h. Subsequently, the subsequent slurry was stable for 1 h and the supernatant was poured out to eliminate impurities. After this purification step, the as-prepared ATP powder was immersed in a solution consisting of 300 mL of deionized water and 200 mL of 30% (*w*/*w*) H_2_O_2_. The suspension underwent magnetic stirring for 6 h and was further subjected to sonication for 30 min at a frequency of 40 kHz. The resulting suspension was centrifuged at 3900 rpm, yielding precipitates, which were subsequently dried for 24 h at 70 °C, and stored for future use (P-ATP). This P-ATP was activated in 150 mL of 1M HCl with continual magnetic stirring for 5 h, followed by a 30 min sonication session to produce A-ATP. The A-ATP was dried for 24 h at 70 °C and stored in a desiccator at room temperature for further use. The P-ATP@Fe_3_O_4_ and A-ATP@Fe_3_O_4_ were prepared by adding a fixed amount (ATP 5: 1 Fe_3_O_4_) of Fe_3_O_4_ nanoparticles into P-ATP and A-ATP, following the same procedure as in the second stage (Figure 1).

### 2.3. Characterization

XRD-6000 X-ray diffractometer (Shimadzu, Kyoto, Japan) running at 40 KV and equipped with Cu Kα = 1.5406 Å radiation was used to perform the X-ray diffraction (XRD) test over a broad angle range of 5–70/2θ^o^. Using the potassium bromide method (sample-to-KBr ratio of 1:100 by mass), The Fourier transform infrared spectroscopy (FTIR) spectra were studied using a Vector 22 infrared spectrophotometer (Bruker, Karlsruhe, Germany) in the 4000–500 cm^−1^ range. Morphological investigations were conducted using a Supra 55 scanning electron microscope (SEM, Zeiss, Jena, Germany). The samples were degassed at 120 °C for three hours, and then low-temperature nitrogen adsorption-desorption studies were carried out using a ASAP 2460 analyzer (Micrometrics, Atlanta, GA, USA). The Brunauer–Emmet–Teller (BET) method was used to examine the pore structure based on the adsorption isotherm.

### 2.4. Batch Degradation

The batch degradation experiment of MB was carried out in a 250 mL conical flask placed in a water bath shaker equipped with a constant temperature oscillator and agitated at 150 rpm in the absence of light. The reaction suspension was prepared by introducing the required catalyst amount (0.1 to 1.0 g/L) into 100 mL of a 100 mg/L MB solution with varying pH values (ranging from 3 to 12). Vibrational agitation was applied for 120 min to attain an adsorption/desorption equilibrium. After achieving equilibrium, 0.25 mL of 30% (*w*/*w*) H_2_O_2_ solution was introduced to initiate the degradation reaction. Throughout the reaction, the concentrations of dye solution were measured at a maximum wavelength of *λ* = 664 nm for each predefined interval and promptly centrifuged at 9000 rpm for 5 min to eliminate the catalysts. Each experiment was conducted in triplicate to ensure reliability. To assess the regeneration capability of ATP@Fe_3_O_4_, the spent composite was separated from the suspension once MB degradation was complete. This regenerated sorbent was then reused for subsequent MB degradation experiments. The degradation rate of MB was calculated using Equation (1):

(1) $$Degradation(\%)=\frac{C_{0}-C_{t}}{C_{0}}\times 100$$

where *C*_0_ and *C_t_* are the concentrations of MB at the initial point and time *t*, respectively, and *C*_0_ − *C_t_* is the final adsorption amount of MB on ATP@Fe_3_O_4_ after degradation.

To further understand the mechanism of degradation, we analyzed the oxidation kinetics of the Fenton-like process and fit the data to the pseudo-first-order kinetic model (Equation (2)):

(2) $$\ln\left(\frac{C_{t}}{C_{0}}\right)=-kt$$

Here, *k* denotes the pseudo-first order rate constant, whereas *C*_0_ and *C_t_* (mgL^−1^) represent the MB concentration at the initial point and time *t*, respectively.

## 3. Result and Discussion

### 3.1. Structural Elucidation

**Figure 2:**
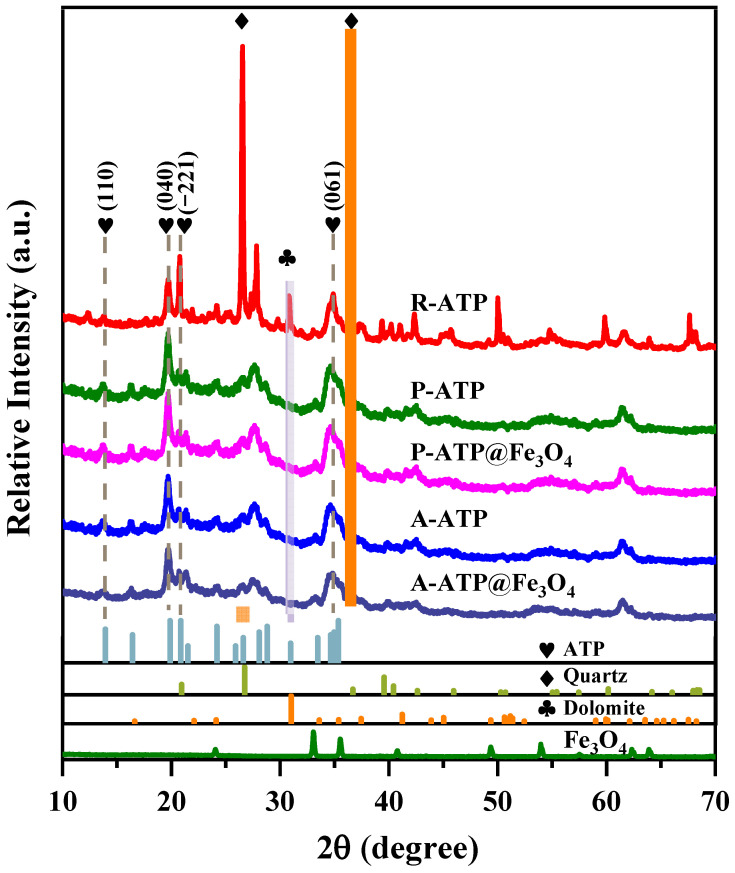
XRD of R-ATP, P-ATP, P-ATP@Fe_3_O_4_, A-ATP, A-ATP@Fe_3_O_4_, ATP, quarts and dolomite.

Figure 2 reveals the powder XRD peak patterns of five samples: R-ATP, P-ATP, P-ATP@Fe_3_O_4_, A-ATP, and A-ATP@Fe_3_O_4_. These peaks of purified ATP (P-ATP) and activated ATP (A-ATP) at 2θ = 9.7° and 19.9° are consistent with the (110) and (040) planes of ATP. The characteristic peaks of mica and dolomite located at 26.7°, 36.7°, and 31.0° were successfully eliminated from P-ATP and A-ATP with the purification of H_2_O_2_ and activation of HCl. The decrease in the number of peaks in the composites shows that the removal of impurities was successfully achieved during the purification and activation processes. The appearance of the weak diffraction peaks of Fe_3_O_4_ at 30.1°, 35.5°, 43.1°, and 56.9° for the respective crystal planes confirms the successful formation of P-ATP@Fe_3_O_4_ and A-ATP@Fe_3_O_4_ composites. The P-ATP@Fe_3_O_4_ and A-ATP@Fe_3_O_4_ exhibited weaker intensities of the ATP peak at 2θ = 19.9°, suggesting the modification of Fe_3_O_4_ on the surface of ATP. The ATP@Fe_3_O_4_ composites still showed distinctive reflections for ATP despite the alteration process, suggesting that the structural integrity of ATP was maintained. Using the Scherer equation, it was possible to calculate the average crystallite size of Fe_3_O_4_ particles on the surface of A-ATP, which came out to be 12.8 nm.

**Figure 3:**
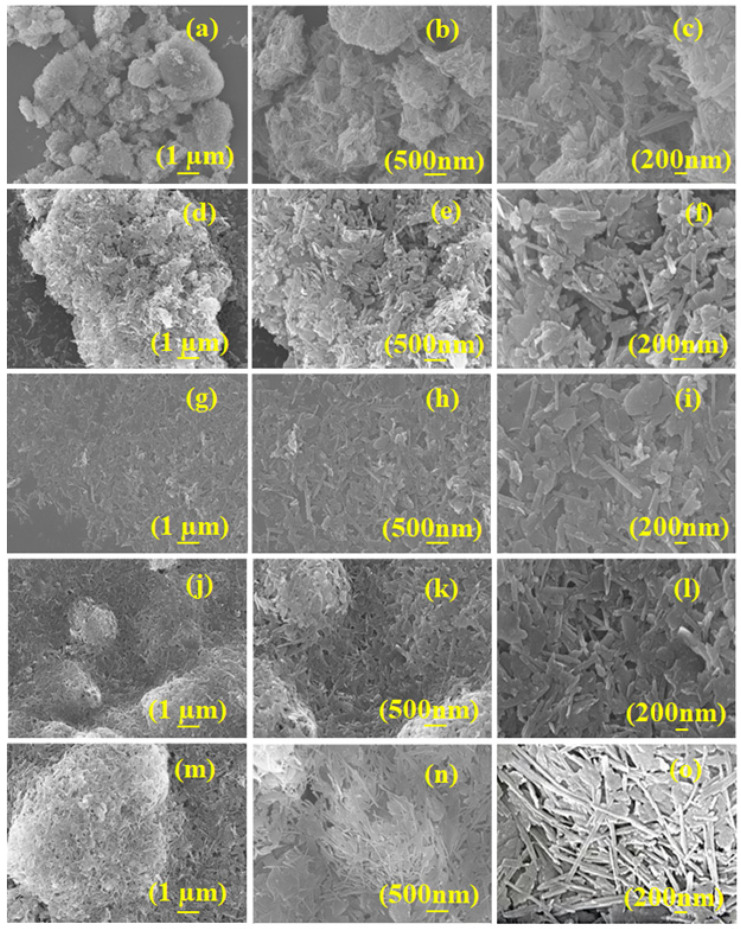
SEM photographs of (**a**–**c**) R-ATP, (**d**–**f**) P-ATP, (**g**–**i**) P-ATP@Fe_3_O_4_, (**j**–**l**) A-ATP, and (**m**–**o**) A-ATP@Fe_3_O_4_ composites.

The ATP rod-like structure exhibits a homogeneous distribution of Fe_3_O_4_ particles, as evidenced by the scanning electron microscopy (SEM) pictures (Figure 3). Based on the SEM pictures, the average size of the Fe_3_O_4_ particles was about 15 nm, which agrees well with the X-ray diffraction (XRD) data. The geometry of RATP (Figure 3a–c) was refined after each step of the treatment, including purification (Figure 3d–f), activation (Figure 3j–l), and composite formation steps (Figure 3g–i,m–o). With the purification and activation of H_2_O_2_ and HCl, the surface impurities decreased significantly, and the rod crystal structure of ATP gradually became obvious. Following the introduction of Fe_3_O_4_ particles, nano-sized particles were seen on the surface of ATP, demonstrating the successful fabrication of ATP-Fe_3_O_4_ composites.

### 3.2. Specific Surface Area Analysis for ATP Composites

**Figure 4:**
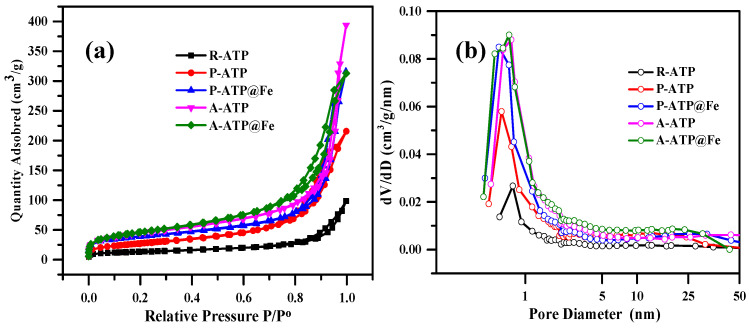
Nitrogen adsorption desorption isotherms (**a**) and pore size distribution (**b**) for R-ATP, P-ATP, P-ATP@Fe_3_O_4_, A-ATP, and A-ATP@Fe_3_O_4_ composites.

At 77K, the adsorbents’ nitrogen (N_2_) adsorption–desorption isotherms were measured (Figure 4a,b). Based on the IUPAC classification, the R-ATP P-ATP, P-ATP@Fe_3_O_4_, A-ATP, and A-ATP@Fe_3_O_4_ isotherms are categorized as Type IV, displaying H3-type hysteresis [32]. The adsorption amount of N_2_ was sharply increased at P/P°, indicating the existence of mesopores and the pores in the adsorbent were narrow slit pores or aggregates of plate-like particles [33]. Table 1 summarizes the variation in surface area and pore size distribution. The surface area and average pore size were elevated after the purification and activation. In particular, it was discovered that the adsorbent containing A-ATP@Fe_3_O_4_ had a substantially larger specific surface area than the other samples with a decreased pore size, demonstrating the successful modification of Fe_3_O_4_ on the surface of P-ATP and A-ATP.

### 3.3. Surface Analysis

**Figure 5:**
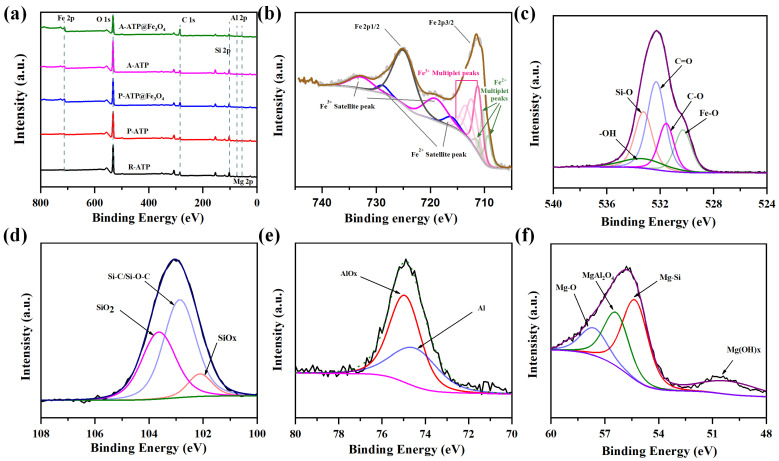
(**a**) XPS comparison of R-ATP, P-ATP, A-ATP, P-ATP@Fe_3_O_4,_ and A-ATP@Fe_3_O_4_, Fe 2p, (**b**) O 1s, (**c**) Si 2p, (**d**), Al 2p, and (**e**) Mg 2p, (**f**) XPS spectra of A-ATP@Fe_3_O_4_ composite.

The XPS test was carried out on five samples to evaluate the effect of purification, activation, and to confirm the formation of composites at subsequent stages. The XPS results for R-ATP, P-ATP, A-ATP, and ATP-composites revealed that the surface composition was within the range of 0 to 800 eV binding energies. Figure 5a shows the presence of various elements, including Al, Mg, Si, O, C, and Fe in R-ATP, P-ATP, A-ATP, and ATP-composites. A-ATP@Fe_3_O_4_ has the highest Fe%, demonstrating the successful incorporation of A-ATP and Fe_3_O_4_ (Table 2). As shown in Figure 5b, the binding energies at 711.3 and 725.6 eV are assigned to Fe 2p3/2 and Fe 2p1/2 of Fe_3_O_4_, respectively. Satellite peaks at 732.8, 719.0, 729.1, and 716.2 eV are ascribed to the charge transfer and shake-up processes of Fe^3+^ and Fe^2+^. The Fe 2p3/2 peak was fitted into seven peaks, including four multiplet peaks for Fe^3+^ (714.7, 713.5, 712.4, and 711.2 eV) and four multiplet peaks for Fe^2+^ (711.5, 710.1, and 709.3) [34,35,36,37]. These results verify the presence of Fe_3_O_4_ on the surface of A-ATP@Fe_3_O_4_. The deconvolution of O 1s spectrum results reveal five types of oxygen functionalities (metallic oxides, C-O, C=O, Si-O, and -OH) with 530.1 eV, 531.7 eV, 532.3 eV, 533.3 eV, and 533.6 eV binding energies, respectively (Figure 5c). The deconvoluted spectrum of Si 2p confirms that it was composed of three isolated peaks (SiO_2_, SiO_x_, and Si-C/Si-O-C) with the corresponding binding energies of 101.9 eV, 102.4 eV, and 103.3 eV (Figure 5d). The deconvolution of the Al 2p spectrum shows it was composed of two distinctive peaks (Al and AlO_x_) with 74.7 eV and 75.2 eV binding energies (Figure 5e). Figure 5f was obtained after the deconvolution of the Mg 2p spectrum to evaluate its functional nature. These results have shown that magnesium is present in the form of MgSiO_3_ (54.2 eV), MgO (57.2 eV), MgAl_2_O_4_ (55.4 eV), and Mg (OH)x (50.7 eV). Furthermore, it can be claimed that the components of A-ATP@Fe_3_O_4_ were composed of aluminum silicate, magnesium silicate, magnesium carbonate, iron-silicate, and iron–oxide with cylindrical (rod-like) morphology and surface-active functional groups carrying a surface charge.

**Figure 6:**
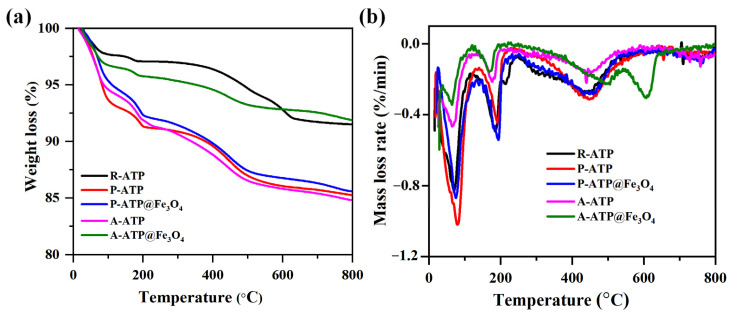
(**a**) Thermogravimetric analysis and (**b**) DTG outcomes of R-ATP, P-ATP, P-ATP@Fe_3_O_4_, A-ATP and A-ATP@Fe_3_O_4_ composites.

To evaluate the thermal stability of R-ATP and ATP-composites, a thermogravimetric analysis (TGA) and a derivative of thermogravimetry (DTG) were performed using a SDT Q-600 analyzer (TA, Newcastle, DE, USA) [38,39]. Samples of 4–6 mg were placed in a coated ceramic crucible and heated at a rate of 10 degrees/min up to 800 °C in a Nitrogen environment with a constant flow rate of 100 mL/min [40]. The TGA and DTG results show that R-ATP, P-ATP P-ATP@Fe_3_O_4_, A-ATP, and A-ATP@Fe_3_O_4_ underwent thermal degradation in three stages: primary (0–200 °C), secondary (200–400 °C), and tertiary (400–800 °C). Raw ATP has the lowest thermal stability with 16% mass loss due to the removal of moisture and volatile compounds in all three stages. P-ATP and P-ATP@Fe_3_O_4_ have shown the same trends as R-ATP but a smaller mass loss of 15%. This weight loss difference may be caused by the removal of mica and dolomite during the purification process (Figure 6a,b). A-ATP and A-ATP@Fe_3_O_4_ show identical weight loss of 12%, which is comparatively smaller than that of R-ATP and P-ATP. This significant change in weight loss occurred due to the effects of acid activation (A-ATP) and composite formation for A-ATP@Fe_3_O_4_. The introduction of metallic oxide enhanced thermal stability by reducing smoke formation and volatile compounds in the primary stage. A-ATP@Fe_3_O_4_ has shown the highest thermal stability of up to 600 °C with 8.9% weight loss in both stages; this inherent stability results from the synergistic effects of the acid activation and composite formation. This thermal stability can be helpful for adsorptive applications due to the availability of numerous surface functionalities.

**Figure 7:**
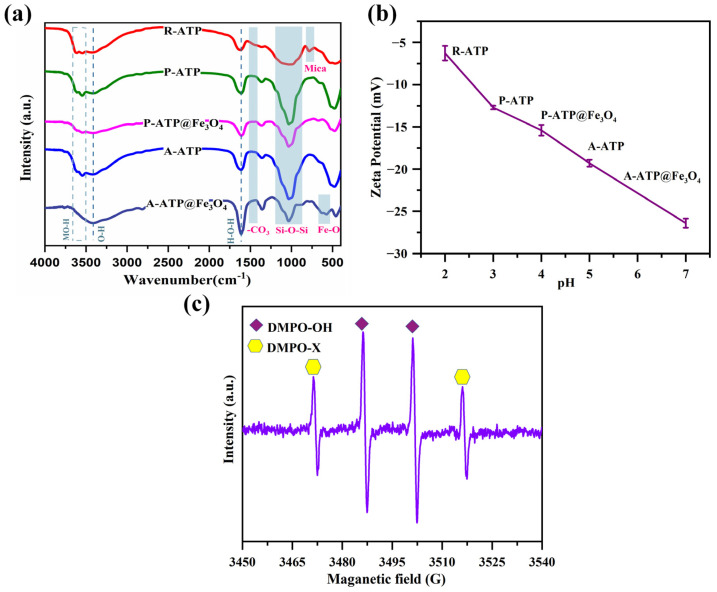
FTIR spectra (**a**) and zeta potential (**b**) of R-ATP, P-ATP, P-ATP@Fe_3_O_4_, A-ATP and A-ATP@Fe_3_O_4_ composites. (**c**) Detection of ·OH radicals in A-ATP@Fe_3_O_4_/H_2_O_2_ heterogeneous Fenton system.

As seen in Figure 7, the FTIR analysis was used to look into the chemical bonds in the R-ATP, P-ATP, P-ATP@Fe_3_O_4_, A-ATP, and A-ATP@Fe_3_O_4_ after the adsorption of MB. The (Al)O-H and (Mg)O-H stretching vibration regions are represented in the range of 3416–3554 cm^−1^. The stretching and bending vibrations of -OH are located at 3430 cm^−1^ and 1650 cm^−1^. The characteristic vibration peaks of carbonate, Si-O-Si, and mica are represented by 1465, 1028 and 783 cm^−1^, respectively. The aromatic ring characteristic peak of the MB was located at 1100–1300 cm^−1^ and appeared in all five samples, confirming the strong adsorption properties of the ATP matrix and the aromatic ring’s absorption. Compared to R-ATP, the characteristic FTIR peaks of mica and carbonate located at 783 and 1465 cm^−1^ were removed in P-ATP and A-ATP, which is consistent with the results presented in the XRD spectra and further verified the purification capacity of H_2_O_2_ and HCl. The characteristic absorption peaks from M-OH and Si-O-Si present at 3416–3554 and 1028 cm^−1^ became sharper in A-ATP, indicating that the activation of HCl can further enhance the purity of ATP. Moreover, the characteristic absorption peak located at 873 cm^−1^ is attributed to the vibration of Fe-O, indicating the successful fabrication of P-ATP@Fe_3_O_4_ and A-ATP@Fe_3_O_4_.

The zeta potential of R-ATP, P-ATP, P-ATP@Fe_3_O_4_, A-ATP, and A-ATP@Fe_3_O_4_ is displayed in Figure 7b to determine their proximal surface charge [41,42,43]. With the treatment and modification of R-ATP by H_2_O_2_, HCl, and Fe_3_O_4_ nanoparticles, the zeta potential of R-ATP, P-ATP, P-ATP@Fe_3_O_4_, A-ATP, and A-ATP@Fe_3_O_4_ gradually becomes negative under neutral conditions. R-ATP shows a zeta potential of −6.3 ± 0.3 mV since it exhibits a structurally negative charge. H_2_O_2_ is an oxidizing agent that can remove inorganic impurities. As shown in the XRD (Figure 2), the characteristic peak of dolomite located at 31.0° was successfully eliminated from P-ATP. The characteristic FTIR peaks of carbonate, located at 1465 cm^−1^, were also removed (Figure 7a), indicating that the surface of dolomite impurities was removed by H_2_O_2_. Dolomite exhibits a positive charge under neutral conditions, and its removal causes P-ATP to exhibit a more negative zeta potential of −12.7 ± 0.2 mV. HCl belongs to a small-sized ionic compound that can enter the unique double electrode layer of ATP, which increases the thickness of the double electrode layer promoting the separation of ATP fibers, and further removes the impurity of ATP on the basis of H_2_O_2_. As demonstrated in the FTIR spectra (Figure 7a), the characteristic absorption peaks of carbonate and quartz at 1465 and 783 cm^−1^ were further removed and the characteristic absorption peaks of M-OH and Si-O-Si at 3416–3554 and 1028 cm^−1^ became sharper. These results indicate that the activation of HCl further enhances the purity of ATP and exposes more structural negative charges, resulting in more negative charges. As shown in the XPS (Figure S1) and XRF data (Table S1), there is no evident change in the proportion of positive ions, such as Si^4+^, Mg^2+^, Al^3+^, and Fe^3+^ in the A-ATP materials with an elevated purity of ATP, indicating that HCl has dissolved the Si^4+^ and metal ions out into a tetrahedra and octahedra of ATP; therefore, leading to a more negative zeta potential of A-ATP to −19.3 ± 0.4 mV. Meanwhile, Fe_3_O_4_ exhibits a negative zeta potential under neutral conditions; therefore, the zeta potentials of P-ATP@Fe_3_O_4_ and A-ATP@Fe_3_O_4_ are slightly lower than those of P-ATP and A-ATP.

An EPR was used to evaluate and measure ROS in the A-ATP@Fe_3_O_4_/H_2_O_2_ system (Figure 7c). 5,5-dimethyl-1-pyrroline 1-oxide (DMPO) was utilized as a trapper and stabilizer for ·OH ROS. The resultant EPR signals, with a ratio of 1:2:2:1, confirm the formation of DMPO-OH, which is an indication of the presence of ·OH radicals [44]. These results confirm that the oxidative degradation of MB using the A-ATP@Fe_3_O_4_/H_2_O_2_ composite is caused by ·OH radicals. Our findings provide discerning evidence regarding the MB breakdown process using A-ATP@Fe_3_O_4_, suggesting the significance of ·OH radicals during the process.

### 3.4. Adsorption and Degradation

**Figure 8:**
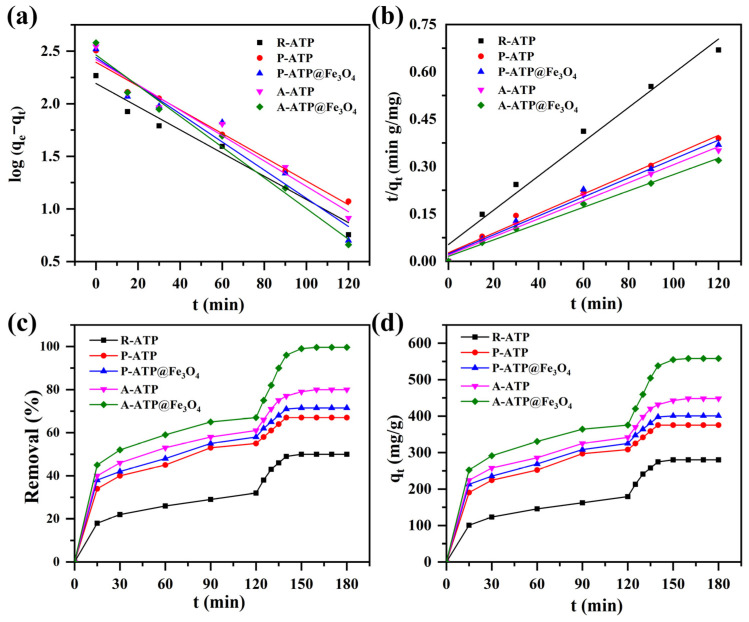
Pseudo-first order (**a**) and pseudo-second order (**b**) kinetic curves, removal efficiency (**c**), and maximum adsorption capacity (**d**) of R-ATP, P-ATP, P-ATP@Fe_3_O_4_, A-ATP and A-ATP@Fe_3_O_4_ composite.

Figure 8a,b demonstrates the adsorption behavior kinetic model of five ATP samples for MB with an initial concentration of 100 mgL^−1^ at pH = 3 and 40 °C. The adsorption equilibrium was achieved in 120 min for all five ATP samples, and then H_2_O_2_ was introduced to achieve the Fenton process for degradation (Figure 8c,d). The maximum removal capacities of R-ATP, P-ATP, P-ATP@Fe_3_O_4_, A-ATP, and A-ATP@Fe_3_O_4_ are 179, 320, 330, 342, and 380 mg/g, respectively. This major change in removal capacities was mainly due to the differences in pore size and well-refined morphological evolution. A-ATP@Fe_3_O_4_ showed the largest BET surface area and the lowest pore size among the samples under investigation. Relative to the values given in the literature, it is noteworthy that the equilibrium time and equilibrium capacity of A-ATP@Fe_3_O_4_ were found to be outstanding and promising [45,46]. In addition, we further studied the adsorption mechanism using the pseudo-first-order [35] given in Equation (3) and the pseudo-second-order given in Equation (4) with corresponding parameters presented in Table 3:

(3) $$\ln(q_{e}-q_{t})=\ln q_{e}-k_{1}t$$

(4) $$\frac{t}{q_{t}}=\frac{1}{k_{2}q_{e}^{2}}+\frac{t}{q_{e}}$$

where *q_e_* (mg·g^−1^) and *q_t_* (mg·g^−1^) are the adsorbed amounts of MB at equilibrium and at time *t* (min), *k*_1_ (min^−1^), and *k*_2_ (mg·g^−1^ min^−1^) are the adsorption rates for the pseudo-first and pseudo-second order models. The adsorption of MB on the five prepared ATP samples was well-fitted for the pseudo-second order, which suggests that the adsorption of MB on porous A-ATP@Fe_3_O_4_ is a chemical-adsorption.

### 3.5. Catalytic Activity

During the degradation process induced by A-ATP@Fe_3_O_4_, the target pollutant MB was first adsorbed on the surface of ATP. When H_2_O_2_ was added after equilibrium, the metal Fe^2+^ ions in ATP and Fe_3_O_4_ activated H_2_O_2_, triggering the production of a large number of ·OH radicals Equation (5). The ·OH radicals attacked the chemical bonds in the MB molecules, eventually degrading the MB into carbon dioxide and water Equation (6). In addition, during the reaction, Fe^3+^ in ATP and Fe_3_O_4_ reacts with H_2_O_2_ to generate Fe^2+^ to achieve the recycling of the catalyst Equations (7) and (8):

(5) $${\mathrm{Fe}}^{2+}+H_{2}O_{2}\to {\mathrm{Fe}}^{3+}+\cdot \mathrm{OH}+{\mathrm{OH}}^{-}$$

(6) $$\mathrm{MB}+\cdot \mathrm{OH}\to {\mathrm{CO}}_{2}+H_{2}O$$

(7) $${\mathrm{Fe}}^{3+}+H_{2}O_{2}\to {\mathrm{Fe}}^{2+}+{\mathrm{HO}}_{2}\cdot +\;H^{+}$$

(8) $${\mathrm{Fe}}^{3+}+{\mathrm{HO}}_{2}\cdot \to {\mathrm{Fe}}^{2+}+O_{2}+H^{+}$$

**Figure 9:**
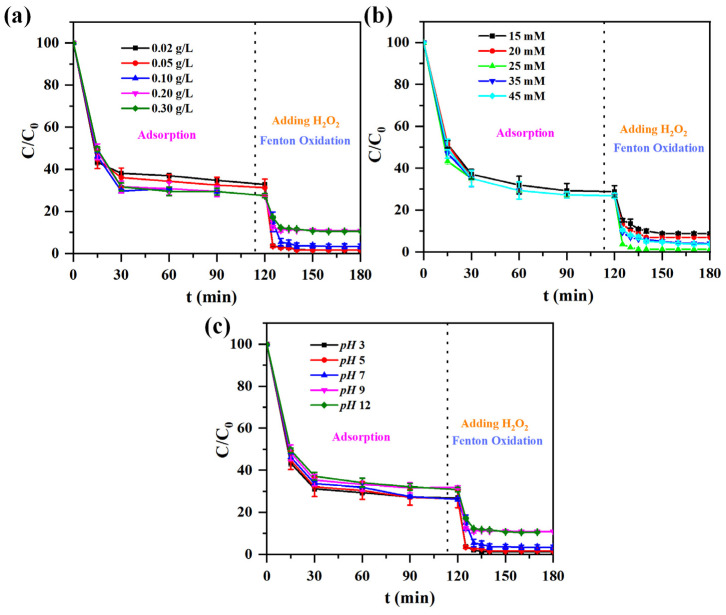
Effect of adsorbate dosage (**a**) H_2_O_2_ dosage, (**b**) pH, and (**c**) on degradation properties induced by A-ATP@Fe_3_O_4_ composite.

Based on the experiment results carried out with an initial MB concentration of 100 mg/L at pH 3 settings, A-ATP@Fe_3_O_4_ exhibit the highest removal efficiency than R-ATP, P-ATP, P-ATP@Fe_3_O_4_, and A-ATP alone after thirty minutes of dark degradation (Figure 8). The impact of adsorbate dosages, content of H_2_O_2_ and pH values on the degradation efficacy of MB using A-ATP@Fe_3_O_4_ was investigated (Figure 9). The dosage of the A-ATP@Fe_3_O_4_ directly depends on the active adsorption sites and catalytic capacity. As shown in Figure 9a, with the increase in the amount of A-ATP@Fe_3_O_4_, the degradation efficiency of MB appears to have a trend of first increasing, then decreasing and achieving the highest adsorption and degradation efficiency with an amount of 100 mg. This is because the lack of A-ATP@Fe_3_O_4_ leads to a lack of adsorption sites and active substances, but a massive overdose of catalyst will consume active radicals.

The impact of H_2_O_2_ dosage on MB removal rates reveals a direct correlation between H_2_O_2_ and MB removal efficacy [47,48,49,50]. At decreasing H_2_O_2_ concentrations, the availability of ·OH radicals decreased, limiting treatment efficacy [51]. Conversely, excessive H_2_O_2_ consumption led to a decrease in the concentration of ·OH radicals generated during the reduction [52,53]. Under the experimental conditions, the dosage of 25 mM H_2_O_2_ produced the best elimination effect (Figure 9b).

To maximize the elimination of MB, careful pH control is essential (Figure 9c) [54,55]. At pH 5, A-ATP@Fe_3_O_4_ demonstrates about 75% MB elimination in 180 min, with degradation rising as pH drops. The elimination of MB is about 99.6% at pH 3, indicating that acidic circumstances are ideal for its effectiveness. The composites demonstrated a significant capacity for MB degradation even with reduced removal rates at higher pH values, suggesting robust catalytic activity over a broad pH range.

The influence of reaction temperature on MB removal was systematically explored under optimal conditions [56,57]. Elevating the reaction temperature from 20 to 50 °C led to a substantial enhancement in the MB removal rate, progressing from 72% to 99.6% (Figure S2). The increase in activity at elevated temperatures can be attributed to the heightened activity of hydroxide ions (·OH) and Fe ions. Nevertheless, it is important to mention that a higher rise in temperature resulted in a decrease in the effectiveness of MB removal. This occurred because the decomposition of H_2_O_2_ reached equilibrium at 40 °C. It is essential to note that for safety reasons, an open container should be employed to dissipate excess heat during the experimental process. In practical applications, maintaining reaction temperatures below 50 °C is recommended, as this range not only ensures safety but also improves the activity and utilization efficiency of the catalyst.

The study underscores that the optimal removal rate of MB is achieved when the H_2_O_2_ dosage is 25 mM under the condition of a 100 mL wastewater volume at a reaction time of 3 h and a temperature of 40 °C.

### 3.6. Degradation Mechanism

**Figure 10:**
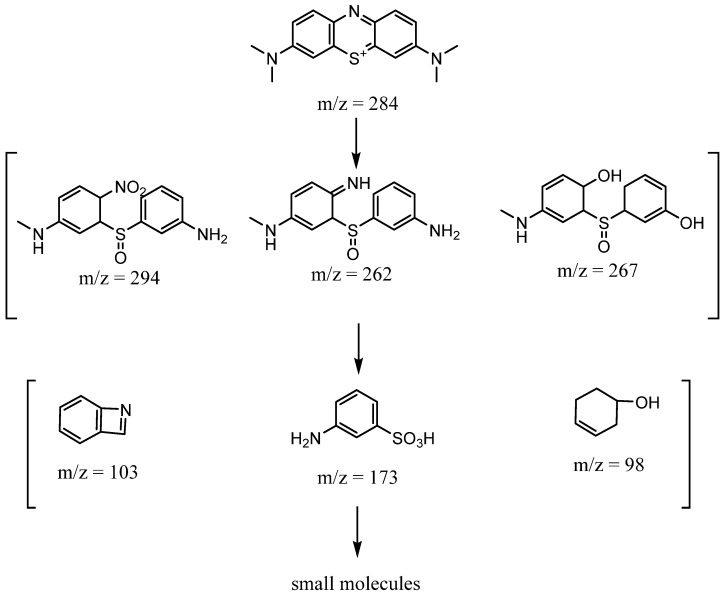
Possible degradation mechanism of MB.

To explore the impact of dissolved Fe concentrations on the degradation of MB, we conducted a heterogeneous Fenton reaction under standard conditions [56]. During the adsorption phase, the ferrous ion concentration gradually rose, peaking at 2.505 mg/L, which is comparable to those reported in the literature [58,59]. Following the addition of H_2_O_2_, the Fe^3+^ concentration decreased to about 0.26 mg/L, resulting in a 99.6% removal rate of MB after 20 min. The dissolved Fe ions originate from Fe in ATP and Fe_3_O_4_, which is also the source of the degradation activity. Further investigation of the degradation products was carried out using liquid chromatography–mass spectrometry (LC–MS). A 20 µL sample of degradation product was introduced into the system using an ESI source. The positive ion mode was selected. The mobile phase consisted of a combination of 0.1 vol% formic acid (A) and (B) acetonitrile (1:5). It was delivered at a flow rate of 0.2 mL/min. Several peaks of varied strengths appeared alongside the MB dye peak, indicating differences in the composition and concentration of the breakdown products. As displayed in Figure S3, the main intermediates of *m*/*z* are 294, 284, 267, 262, 173, 103, and 98, and the possible degradation route is shown in Figure 10 [46,47].

### 3.7. Reusability

**Figure 11:**
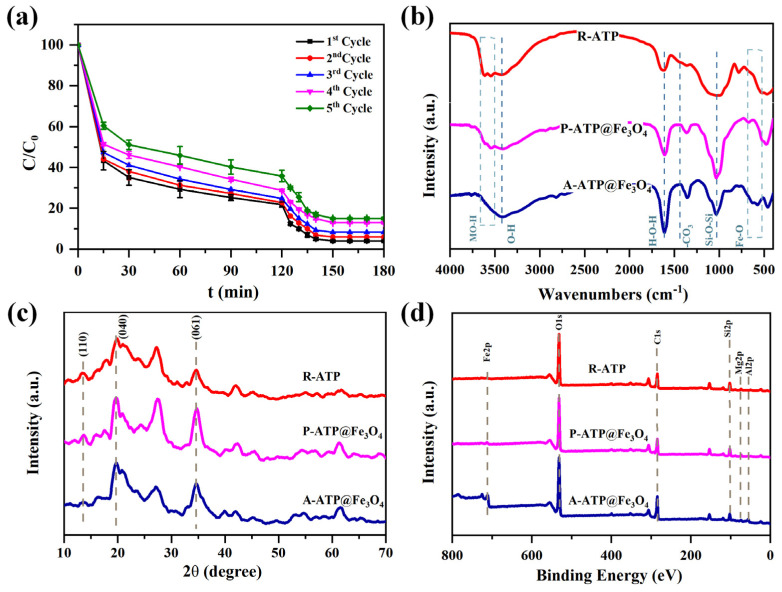
(**a**) Reusability of A-ATP@Fe_3_O_4_; FTIR, (**b**) XRD, and (**c**) XPS (**d**) spectra of R-ATP, P-ATP@Fe_3_O_4_, and A-ATP@Fe_3_O_4_ after degradation.

The recycling of A-ATP@Fe_3_O_4_ was achieved after each degradation. The composites were recovered by ethanol until neutralization. Subsequently, the material was dried in an oven at 70 °C for 12 h. The morphology of the A-ATP@Fe_3_O_4_ composite after degradation was characterized using SEM. As can be seen from Figure S4, the A-ATP@Fe_3_O_4_ composite has greatly maintained its original morphology. Upon reaching the 5th cycle, a slight decrease of 5–10% of the maximum degradation capacity was observed (Figure 11a). As shown in Figure 11b, the functional groups were retained ~100% after recycling, which means that the degraded dye was completely washed away after the Fenton degradation process. This retention was convincingly witnessed and confirmed because even the intensity of the FTIR spectrum remains constant before and after degradation for each adsorbate sample. The relative decrease in full-width half maximum (FWHM) of the XRD spectrum R-ATP, P-ATP@Fe_3_O_4_, and A-ATP@Fe_3_O_4_ after the Fenton degradation process implies that the concentration of silicates was lower down and the arrangement of the lattice remained persistent (Figure 11c). Furthermore, the XPS and XRF survey analysis revealed that the characteristic groups on the surface of R-ATP, P-ATP@Fe_3_O_4_, and A-ATP@Fe_3_O_4_ remained unchanged (Figure 11d and Figure S5 and Table S2). The FTIR spectra, XRD patterns, and XPS spectra remained constant before and after the fifth cycle degradation, demonstrating the stability of the attapulgite-Fe_3_O_4_ composite.

### 3.8. Method Comparison

A-ATP@Fe_3_O_4_ efficacy as a catalyst for eliminating organic dyes was evaluated in comparison to other catalysts that contained iron (Table 4) [58,59,60,61]. For the elimination of organic dyes, many documented Fe-containing catalysts call for greater H_2_O_2_ and catalyst concentrations in addition to extra energy sources like light. This restricts the usefulness of these catalysts and leads to an increase in energy consumption. Additionally, it is challenging to concentrate contaminants on these catalysts’ surfaces due to their limited adsorption capability. Moreover, the Fenton catalysts that are used nowadays are synthetic and necessitate a variety of intricate production procedures. On the other hand, natural attapulgite minerals, which are easily accessible for pretreatment and industrial production, served as the catalysts in this investigation. A-ATP@Fe_3_O_4_ is an outstanding alternative that is being used as a heterogeneous Fenton-like catalyst because of its exceptional degradation efficiency in a short time.

## 4. Conclusions

A natural porous ATP clay was purified and activated, followed by surface modification with Fe_3_O_4_ via co-precipitation. The produced ATP@Fe_3_O_4_ was used for the degradation of MB and the removal rate was achieved at 99.6%, with an adsorption–coupled Fenton oxidation removal mechanism. The adsorption and degradation of MB on ATP followed the pseudo-second-order kinetic model. After five cycles, the removal rate remained above 90%, demonstrating that ATP@Fe_3_O_4_ Fenton-like catalysts have high degradation efficiency and durability. At the same time, this study paves the way for the high-value use of low-grade ATP and offers a novel concept for designing wastewater treatment materials based on an adsorption-coupled Fenton oxidation strategy.

## Figures and Tables

**Table 1:** Nitrogen adsorption and desorption outcomes for various types of ATP composites.

No.	Sample	Surface Area (m^2^/g)	Total Pore Volume (mL/g)	Average Pore Size (nm)
1	R-ATP	46.15	0.14	13.53
2	P-ATP	97.13	0.32	11.58
3	P-ATP@Fe_3_O_4_	133.08	0.46	15.19
4	A-ATP	156.73	0.57	14.76
5	A-ATP@Fe_3_O_4_	163.66	0.47	10.55

**Table 2:** XPS survey analysis of ATP and ATP-composites before adsorption.

% Atomic of Elements						
Elements	% C	% O	% Al	% Si	% Mg	% Fe
R-ATP	20.99	55.77	5.45	13.77	2.64	1.38
P-ATP	15.53	56.44	6.38	16.84	2.77	2.04
P-ATP@Fe_3_O_4_	8.94	58.44	6.44	15.81	3.54	6.53
A-ATP	15.10	56.56	5.56	16.56	2.10	3.05
A-ATP@Fe_3_O_4_	6.93	59.89	6.60	15.55	3.76	7.61

**Table 3:** Kinetic parameters for the adsorption of MB on R-ATP, P-ATP, P-ATP@Fe_3_O_4_, A-ATP, and A-ATP@Fe_3_O_4_.

Samples	*q_e,exp_* (mg/g)	Pseudo-First Order			Pseudo-Second Order		
		*q_e_* (mg/g)	*k*_1_	R^2^	*q_e_*	*k*_2_	R^2^
R-ATP	179.34 ± 1.45	155.96	−0.0048	0.955	185.19	0.0006	0.981
P-ATP	320.35 ± 1.78	246.04	−0.0048	0.967	322.58	0.0004	0.985
P-ATP@Fe_3_O_4_	330.86 ± 1.56	272.77	−0.0058	0.949	333.33	0.0004	0.986
A-ATP	342.39 ± 1.83	263.69	−0.0053	0.961	344.83	0.0004	0.989
A-ATP@Fe_3_O_4_	380.65 ± 1.89	291.21	−0.0063	0.977	384.62	0.0004	0.993

**Table 4:** Comparative study of the removal efficacy of organic dyes by loading Iron composites.

Catalyst	Reaction Conditions				Degradation Efficiency	Mechanism	Refs
	Dyes	[Dyes] (mg L^−1^)	[Catalyst] (g L^−1^)	[H_2_O_2_] (mM)			
3D γ-Fe_2_O_3_@ZnO	CIP	15.0	1.25	35	18.30% (30 min)	Photocatalysis	[58]
Fe^2+/^H_2_O_2_	MB	50	1.5	35.3	99% (40 min)	Fenton-like	[59]
Fe_3−x_Ti_x_O_4_	MB	100	3.0	300	74.40% (100 min)	Adsorption Fenton-like	[60]
Fe_2_(MoO_4_)_3_	MB	100	1.0	18	91.3% (30 min)	Photo-Fenton-like	[61]
AATP@Fe_3_O_4_	MB	100	0.1	100	99.6% (20 min)	Adsorption Fenton-like	This work

## Data Availability

Data are contained within the article and Supplementary Materials.

## Supplementary Materials

The following supporting information can be downloaded at: https://www.mdpi.com/article/10.3390/ma17112615/s1, Figure S1: O1s and Fe 2p XPS comparison of R-ATP (a,b), P-ATP (c,d) P-ATP@Fe_3_O_4_ (e,f) A-ATP (g,h) A-ATP@Fe_3_O_4_ composite (i,j), Table S1: XRF analysis of R-ATP, P-ATP, P-ATP@Fe_3_O_4_, A-ATP and A-ATP@Fe_3_O_4_ composite, Figure S2: Effect of temperature on adsorption & degradation process of R-ATP, P-ATP@Fe_3_O_4_, A-ATP, and A-ATP@Fe_3_O_4_ composite, Figure S3: Main degradation intermediates of MB determined by Mass spectra, Figure S4: Representative SEM images of A-ATP@Fe_3_O_4_ before (a–c) and after (d–f) degradation, Figure S5: Fe 2p (a), O1s (b), Si 2p (c), Al 2p (d) and Mg 2p (e) XPS spectra of A-ATP@Fe_3_O_4_ after degradation, Table S2. XPS survey analysis of R-ATP, P-ATP, P-ATP@Fe_3_O_4_, A-ATP, and A-ATP@Fe_3_O_4_ composites after degradation.
