# Supplementary material for: Fenton-like Degradation of Methylene Blue on Attapulgite Clay Composite by Loading of Iron–Oxide: Eco-Friendly Preparation and Its Catalytic Activity
Source: Materials (Basel). 2024 May 29;17(11):2615. doi: 10.3390/ma17112615 (PMC11174012; doi:10.3390/ma17112615)
Supplement: Supplementary file 1 [file materials-17-02615-s001.zip › materials-2981599-supplementary.pdf]

Fenton-like Degradation of Methylene Blue on Attapulgite Clay Composite by Loading of Iron–Oxide: Eco-Friendly Preparation and Its Catalytic Activity

Naveed Karim [1], Tin Kyawoo [1], Chao Jiang [1], Saeed Ahmed [2], Weiliang Tian [3], Huiyu Li [1,*] and Yongjun Feng [1,3,*]

[1] State Key Laboratory of Chemical Resource Engineering, Beijing Engineering Center for Hierarchical Catalysts, Beijing University of Chemical Technology, No. 15 Beisanhuan East Road, Chaoyang District, Beijing 100029, China; karimnaveed999@gmail.com (N.K.); tinkyawoo.mu@gmail.com (T.K.); 15222443969@163.com (C.J.)

[2] Department of Chemistry, University of Chakwal, Chakwal 48800, Pakistan; saeed.ahmed@uoc.edu.pk

[3] College of Chemistry and Chemical Engineering, Tarim University, Alar 843300, China; 120100037@taru.edu.cn

* Correspondence: huiyuli@buct.edu.cn (H.L.); yjfeng@mail.buct.edu.cn (Y.F.)

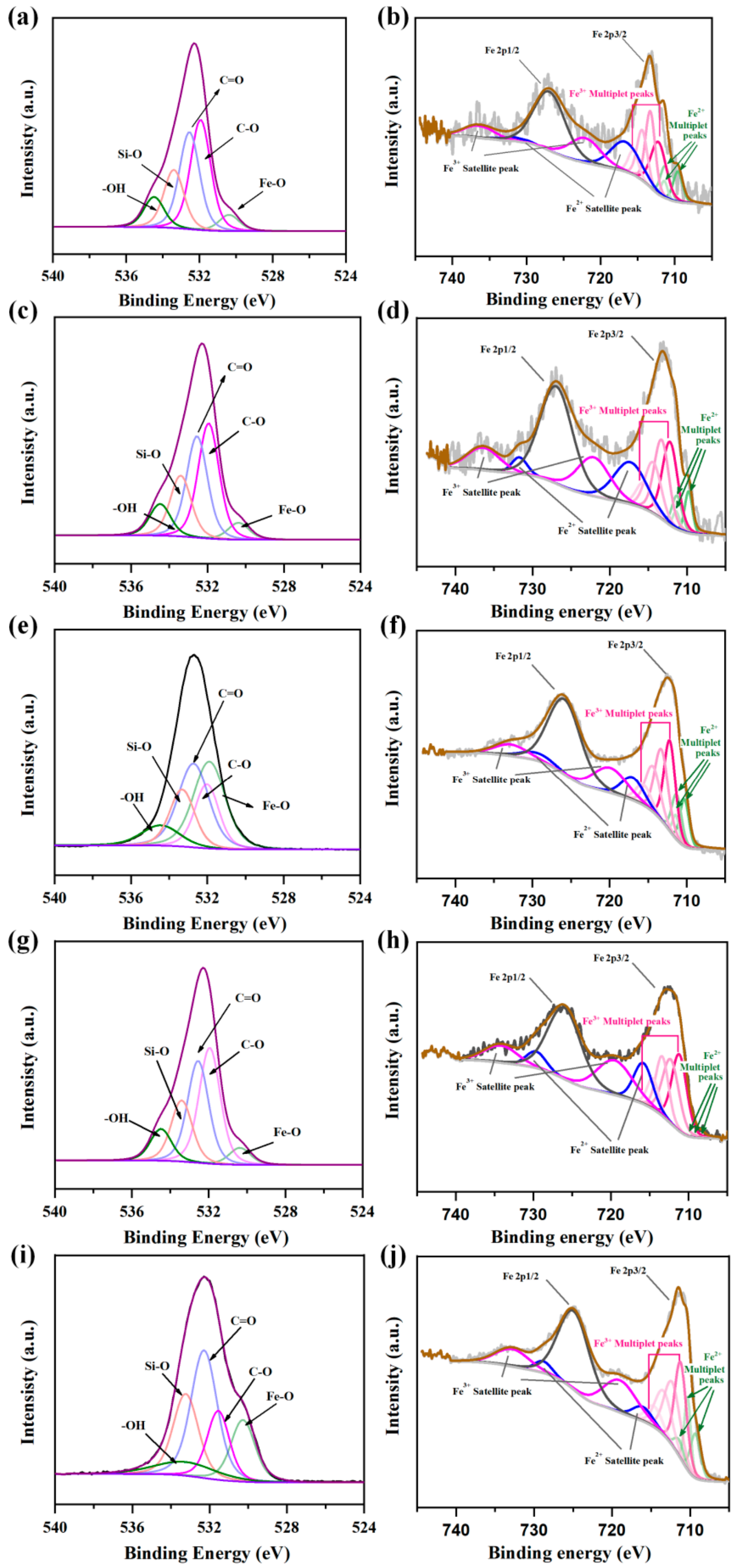

Figure S1. O1s and Fe 2p XPS comparison of R-ATP (a-b), P-ATP (c-d) P-ATP@Fe_3O_4 (e-f) A-ATP (g-h) A-ATP@Fe_3O_4 composite (i-j).

Table S1. XRF analysis of R-ATP, P-ATP, P-ATP@Fe_3O_4, A-ATP and A-ATP@Fe_3O_4 composite.

Elements	SiO_2 (%)	Al_2O_3 (%)	Fe_2O_3 (%)	CaO (%)	MgO (%)	K_2O (%)	TiO_2 (%)
R-ATP	48.11	14.05	14.93	4.56	5.72	6.59	1.21
P-ATP	53.5	14.46	13.42	1.34	7.27	5.28	0.317
P-ATP@Fe_3O_4	50.25	12.75	19.41	0.74	6.65	5.59	0.51
A-ATP	48.96	15.26	15.20	4.27	6.72	7.01	1.14
A-ATP@Fe_3O_4	47.71	12.85	25.28	0.13	5.58	5.16	0.44

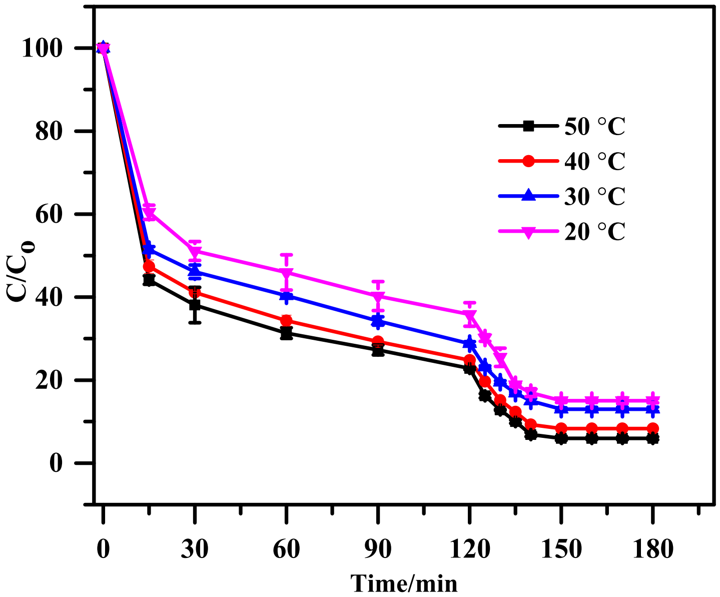

Figure S2. Effect of temperature on adsorption & degradation process of R-ATP, P-ATP@Fe_3O_4, A-ATP, and A-ATP@Fe_3O_4 composite.

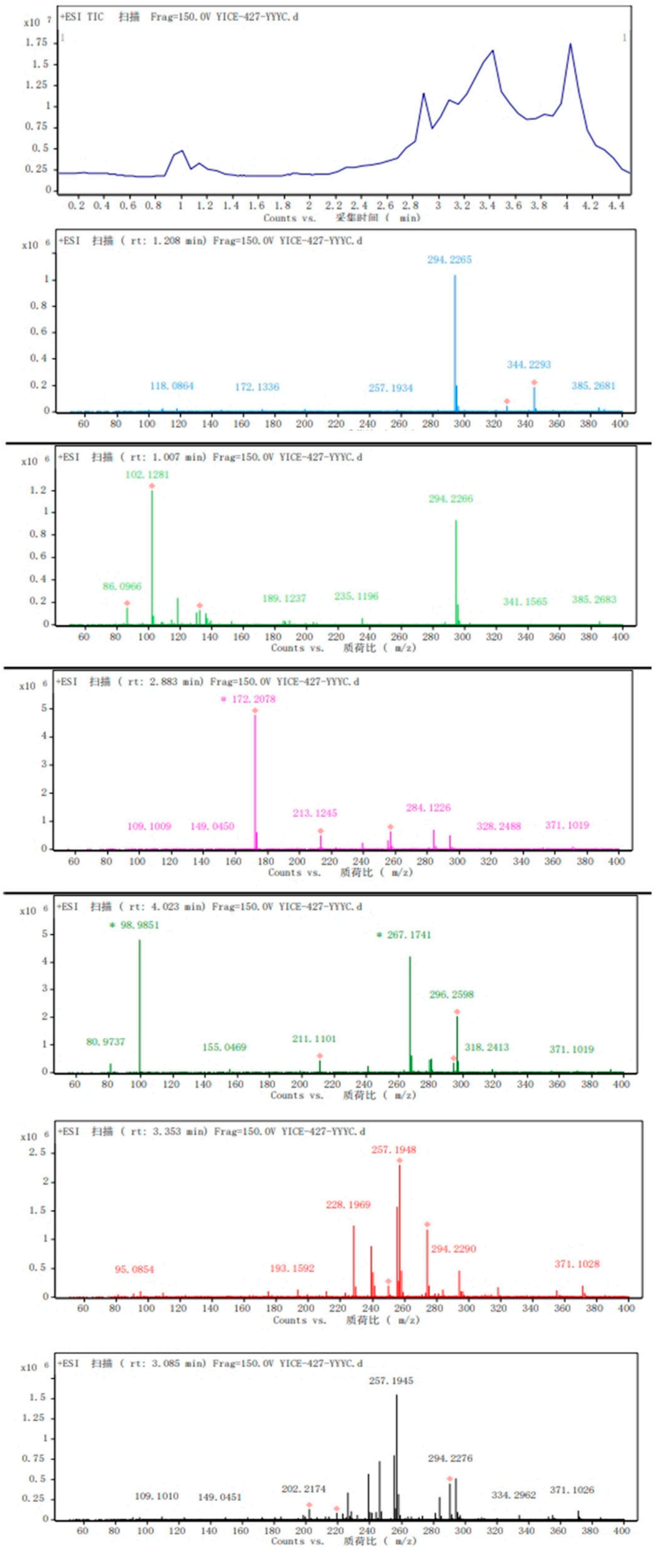

Figure S3. Main degradation intermediates of MB determined by Mass spectra.

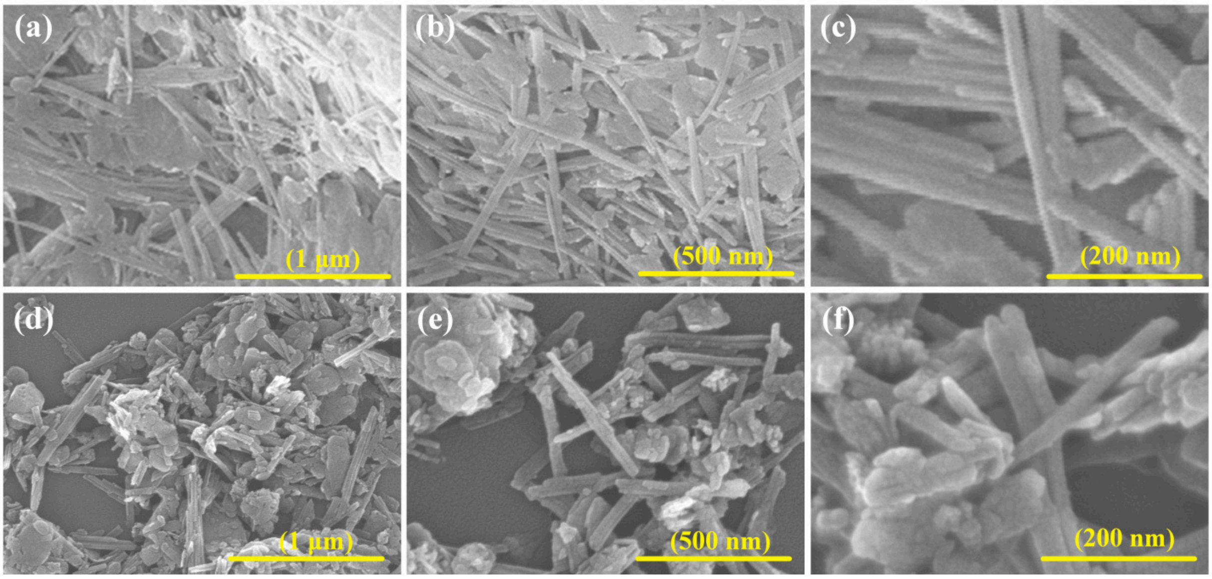

Figure S4. Representative SEM images of A-ATP@Fe_3O_4 before (a-c) and after (d-f) degradation.

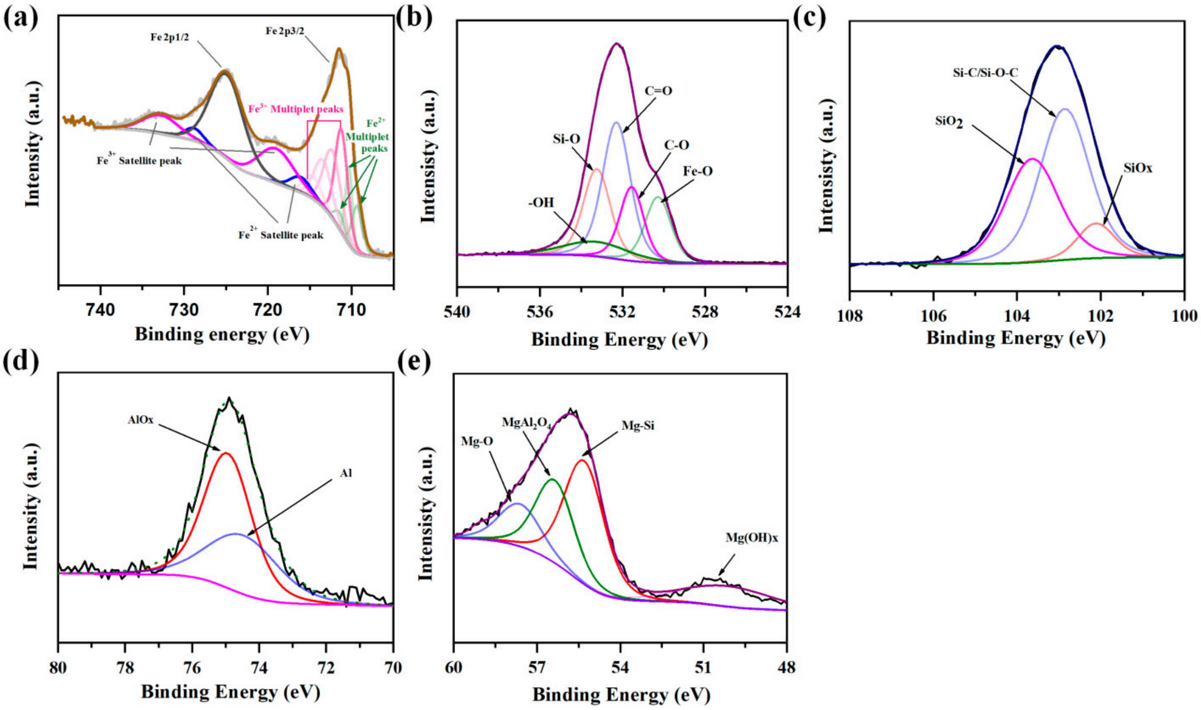

Figure S5. Fe 2p (a), O1s (b), Si 2p (c), Al 2p (d) and Mg 2p (e) XPS spectra of A-ATP@Fe_3O_4 after degradation.

Table S2. XPS survey analysis of R-ATP, P-ATP, P-ATP@Fe_3O_4, A-ATP, and A-ATP@Fe_3O_4 composites after degradation.

% Atomic of Elements						
**Elements**	**% C**	**% O**	**% Al**	**% Si**	**% Mg**	**% Fe**
**R-ATP**	25.59	55.35	5.58	6.12	5.04	1.32
**P-ATP**	18.07	58.46	4.71	9.34	7.11	1.68
**P-ATP@Fe_3O_4**	14.3	59.09	5.02	9.89	7.34	4.36
**A-ATP**	15.96	58.17	5.61	9.74	7.69	2.83
**A-ATP@Fe_3O_4**	12.82	59.24	5.05	9.45	7.23	6.21
